# Supplementary material for: Optimization of differentiation and transcriptomic profile of THP-1 cells into macrophage by PMA
Source: PLoS One. 2023 Jul 17;18(7):e0286056. doi: 10.1371/journal.pone.0286056 (PMC10351730; doi:10.1371/journal.pone.0286056)
Supplement: S1 File — (ZIP) [file pone.0286056.s001.zip › supporting files/RNA-seq report.zip!/RNA-seq report/1_QC_result/ErrorRate/induce-1_error.pdf]

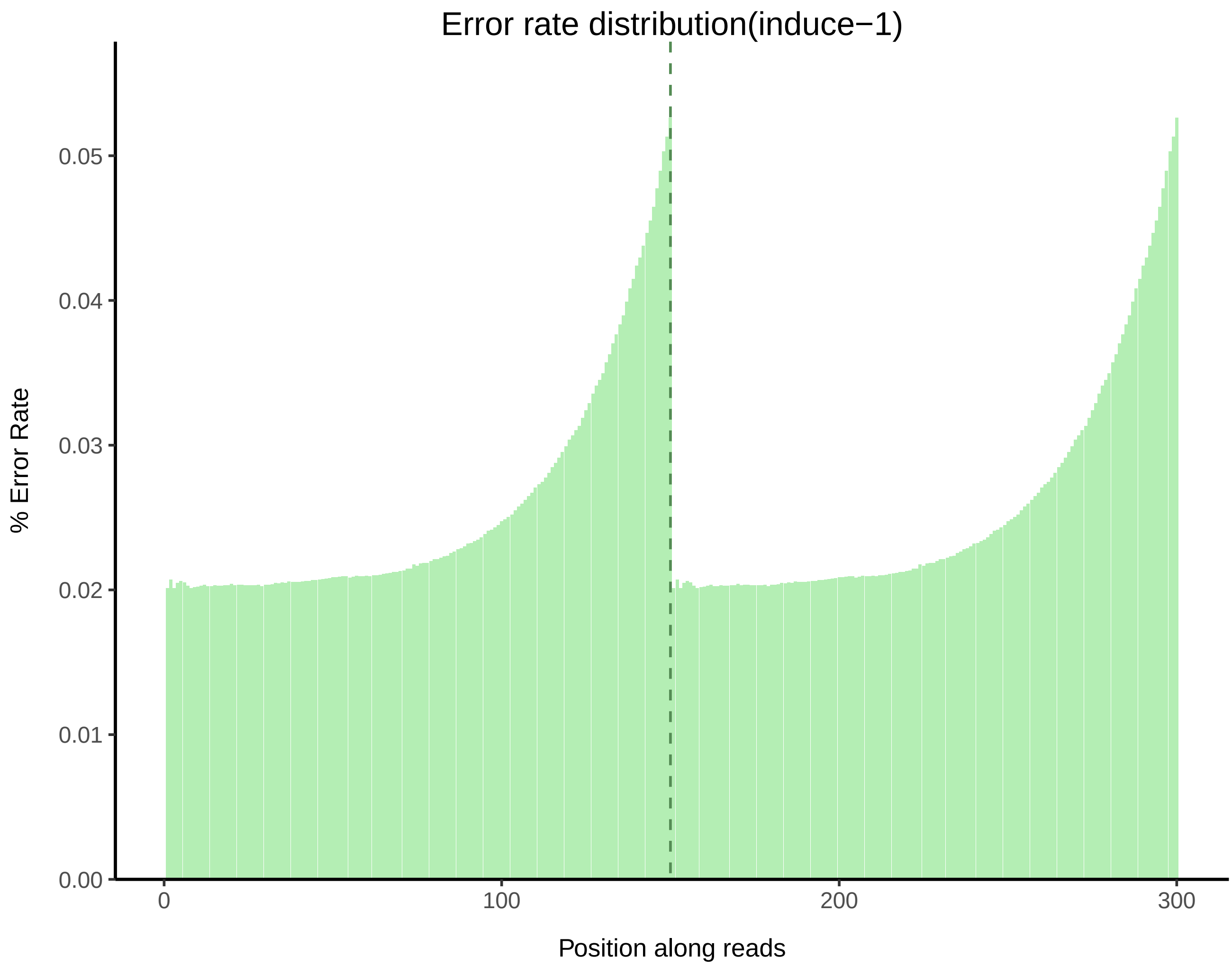
Error rate distribution(induce−1)
% Error Rate
0.05
0.04
0.03
0.02
0.01
0.00
0
100
200
300
Position along reads
